# What drugs modify the risk of iatrogenic impulse-control disorders in Parkinson’s disease? A preliminary pharmacoepidemiologic study

**DOI:** 10.1371/journal.pone.0227128

**Published:** 2020-01-07

**Authors:** Nakyung Jeon, Marco Bortolato

**Affiliations:** 1 College of Pharmacy, Chonnam National University, Gwang-ju, Republic of Korea; 2 Department of Pharmacology and Toxicology, College of Pharmacy, University of Utah, Salt Lake City, Utah, United States of America; University of Toronto, CANADA

## Abstract

**Introduction:**

Parkinson’s disease (PD) patients treated with pramipexole (PPX) and ropinirole (ROP) exhibit a higher risk of developing impulse control disorders (ICDs), including gambling disorder, compulsive shopping, and hypersexuality. The management of ICDs in PD is challenging, due to the limited availability of effective therapeutic alternatives or counteractive strategies. Here, we used a pharmacoepidemiological approach to verify whether the risk for PPX/ROP-associated ICDs in PD patients was reduced by drugs that have been posited to exert therapeutic effects on idiopathic ICDs–including atypical antipsychotics (AAs), selective serotonin reuptake inhibitors (SSRIs), and glutamatergic modulators (GMs).

**Methods:**

To quantify the strength of the associations between PPX/ROP and other medications with respect to ICD risk, odds ratios (ORs) were calculated by multivariable logistic regression, adjusting for age, gender, marital status race, psychiatric comorbidities, and use of cabergoline and levodopa.

**Results:**

A total of 935 patients were included in the analysis. Use of GMs, SSRIs, and AAs was not associated with a decreased ICD risk in PD patients treated with PPX/ROP; conversely, ICD risk was significantly increased in patients treated with either GMs (Adjusted Odds Ratio, ORa: 14.00 [3.58–54.44]) or SSRIs (ORa: 3.67 [1.07–12.59]). Results were inconclusive for AAs, as available data were insufficient to compute a reliable ORa.

**Conclusions:**

These results suggest that some of the key pharmacological strategies used to treat idiopathic ICD may not be effective for ICDs associated with PPX and ROP in PD patients. Future studies with larger cohorts are needed to confirm, validate, and extend these findings.

## Introduction

A large body of evidence has documented that Parkinson’s disease (PD) patients treated with dopamine replacement therapies (DRTs) exhibit a significantly greater risk of developing behavioral complications. [1,2] Pramipexole (PPX) and ropinirole (ROP), two of the best dopaminergic agonists for motor symptom management in early PD stages, [3,4] have been particularly associated with impulse control disorders (ICDs). [5–7] This umbrella term refers to a group of psychiatric conditions characterized by high risk-taking propensity, such as gambling disorder, compulsive shopping, compulsive sexual behavior, and binge eating. Although some of these entities have been moved to different diagnostic categories in the fifth edition of the Diagnostic and Statistical Manual for Mental Disorders (DSM-5) and the upcoming eleventh version of the International Classification of Diseases (ICD-11), this nomenclature is still largely used to refer to the impulsive/compulsive complications of DRTs in PD. [8]

ICDs often compound the severe social and financial burden experienced by PD patients, [1] and their management often poses serious clinical challenges. The best-validated therapeutic strategy for these problems is the dose reduction or discontinuation of PPX and ROP. [9] However, tapering these drugs is not always successful in alleviating ICDs, and can lead to the exacerbation of PD motor symptoms [10,11]. Furthermore, PPX/ROP discontinuation often leads to dopamine agonist withdrawal syndrome (DAWS), characterized by anxiety, panic attacks, diaphoresis, fatigue, dysphoria, and depression; these symptoms further impair functioning, cause significant distress, and are often refractory to other DRTs. [10] Unfortunately, no FDA-approved alternative treatments are currently available for these patients, underscoring the need for novel therapies to either prevent iatrogenic ICDs or mitigate their severity.

Over the past decade, several preliminary clinical trials have identified new putative therapeutic strategies for idiopathic ICDs and compulsive behaviors. Most of the research has pointed to five distinct categories of drugs that may have some efficacy as therapies for these disorders or related symptoms; selective serotonin reuptake inhibitors (SSRIs); [12–14] atypical antipsychotics (AAs); [15,16] mood stabilizers (MSs) such as lithium, carbamazepine, and valproic acid; [17,18] opioid receptor antagonists (ORAs); [19,20] and glutamatergic modulators (GMs), including amantadine, memantine, topiramate, and N-acetylcysteine have emerged as potential ICD treatments. [21,22] While some of these therapies have been anecdotally used in PPX/ROP-associated ICDs, no conclusive evidence is available on their efficacy in reducing these iatrogenic complications. [23,24]

Given the clinical and symptomatic similarities between ICDs secondary to PPX/ROP and their idiopathic counterparts, we hypothesized that the risk of drug-induced ICDs in PD patients may be mitigated by the same drugs that have been posited to exert therapeutic properties for idiopathic ICDs. To address this possibility, we performed a retrospective matched cohort study aimed at assessing whether each of the aforementioned drug classes may reduce the risk of ICD in PD patients treated with either PPX or ROP.

## Materials and methods

The present retrospective cohort study was conducted utilizing the institutional electronic medical records at the University of Utah (UU) Healthcare system. The UU Institutional Review Board approved this study by expedited review under a waiver of informed consent.

### Data source

Clinical and administrative data for 7,375 adult patients with at least one ICD 9/10-CM diagnostic code for PD (ICD-9-CM: 332, 332.0; ICD-10-CM: G20) between Oct 1994 and Feb 2019 were obtained from the University of Utah Enterprise Data Warehouse (UUEDW). The data contained diagnostic and prescription information from inpatient and outpatient records, along with demographic information.

### Inclusion/exclusion criteria and cohort entry

PD patients who received PPX or ROP at or after the first PD diagnosis were considered eligible. Cohort entry was defined as the date when a patient received the first PPX or ROP prescription. To ensure sufficient baseline and follow-up time to capture patient health information (including ICD development), we included only established patients who had one or more non-emergency hospital visits at least six months before and two years after the first PPX or ROP prescription. Patients with a diagnostic code for ICD (ICD-9-CM: 312.3X or ICD-10-CM: F63.X) at or before cohort entry were excluded.

### Identification of the initiation of GMs, SSRIs, AAs, MSs, and ORAs concomitant with PPX/ROP use

Prescription records of GMs, SSRIs, AAs, MSs and ORAs were identified to develop five individual cohorts and assess drug class effects on ICD risk individually. GMs included acamprosate, acetylcysteine, amantadine, memantine, and topiramate. SSRIs included citalopram, escitalopram, fluoxetine, fluvoxamine, paroxetine, and sertraline. AAs included asenapine, clozapine, quetiapine, olanzapine, paliperidone, iloperidone, lurasidone, risperidone, ziprasidone, and aripiprazole. MSs included lithium, carbamazepine, and valproic acid. Lastly, ORAs included naltrexone and nalmefene.

In each cohort, all patients were assigned to one of two groups defined by exposure to the five main drug classes. We excluded patients who had a prescription record of GMs, SSRIs, AAs, MSs, and ORAs prior to their cohort entry or did not continue to receive PPX/ROP at the time of the first exposure to the five drug classes.

### Index date, matching, and follow-up

For patients treated with GMs, SSRIs, AAs, MSs, and ORAs, the index date was defined as the first prescription date of these drugs. To establish a non-exposed group, for each exposed patient we selected up to 3 patients who never received the drug of interest, matching (with replacement) by age at cohort entry (± 1 year) and gender. Furthermore, we counted the number of days between the date of cohort entry (PPX/ROP initiation) and the index date (GM, SSRI, AA, MS and ORA initiation) for each exposed patient. Matching patients were followed up after the same number of days after the date of cohort entry to allow for the same duration of PPX/ROP treatment between groups at baseline. Baseline period was defined as the period between the cohort entry and the index date. Only non-exposed patients who were on PPX/ROP at the index datewere eligible for matching to an exposed patient.

### Analysis

We constructed three individual statistical models to assess associations between ICD incidence and GMs, SSRIs, and AAs. Of note, MSs and ORAs were only prescribed in less than 1% of the study population and the analyses of these two drug categories were not further pursued due to their limited statistical power. ICD incidence was estimated as the number of patients with diagnostic code definitions for ICDs per 100 PD patients taking PPX/ROP who met inclusion/exclusion criteria.

To contrast the association of GM, SSRI, and AA use with ICD incidence, we estimated ICD incidences following exposure to each of the three drug classes among PPX/ROP receiving PD patients. The incidences were then compared with non-users of GMs, SSRIs, and AAs in separate models using conditional logistic regression models. Crude odds ratios (OR) along with 95% confidence intervals (CIs) were calculated to estimate the effect between the use of the psychiatric medications and the incidence of ICDs. We also adjusted the models by baseline characteristics if sample size allowed.[25] Patient characteristics considered for adjustment included: patient age at index date; gender; marital status; race; use of cabergoline or levodopa [26]; and psychiatric comorbidities (such as Delirium/Dementia/Amnestic/Other Cognitive Disorders, Depressive Disorders, Bipolar Disorders, Schizophrenia and Other Psychotic Disorders, and Alcohol or Substance-Related Disorders). [27–30] The comorbid conditions were identified by encounters with respective ICD–9/10–CM codes (available in S1 Table) up to 1 year prior to or at index date. The three main drug classes were not considered to adjust for each other. Variables were reported as percentages and means along with standard deviations (SD). Differences in baseline characteristics were assessed using the X^2^-test, the exact calculation of the Fisher exact test, and 2-sample t-test as appropriate.

An alpha value of 0.05 was the significance level considered for all statistical tests. Analyses were generated using SAS software 9.4. Copyright, SAS Institute Inc. SAS and all other SAS Institute Inc. product or service names are registered trademarks or trademarks of SAS Institute Inc., Cary, NC, USA.

## Results

### Study population

Among 7,375 PD patients in the source study population, 1,791 patients (24.3%) had a PPX/ROP prescription at or after the first PD diagnosis. Of these, 940 (52.5%) patients had one or more non-emergency in- or outpatient visits at least six months before and two years after the cohort entry. Finally, 5 patients who had been diagnosed with ICDs at or before the first PPX/ROP prescription were excluded.

We assessed the utilization of the main five classes of drugs that have been associated with potential therapeutic effects in ICDs. Among the 935 patients included in our analysis, the most commonly prescribed drugs were GMs (505 patients, 54.0%) followed by SSRIs (473 patients, 50.5%) and AAs (237 patients, 25.3%). After excluding patients who were either prevalent users at cohort entry or no longer received PPX/ROP at index date, 264 (52.3%), 205(43.3%), and 105 patients (44.3%) were remained in analyses who were newly prescribed with GMs, SSRIs, and AAs, respectively, at or after PPX/ROP initiation. Less than 1% of 935 patients initiated MSs or ORAs. As shown in Table 1, amantadine was predominant (227/264, 86.0%) among four types of the GMs considered in our analysis, followed by memantine (41/264, 15.5%). Among SSRI new users, es/citalopram (149/205, 72.7%), sertraline (61/205, 29.8%), and fluoxetine (29/205, 14.1%) were the most frequently prescribed. Lastly, 91.4% of AA new users received quetiapine (96/105). Olanzapine was the second most prevent prescriptions (19/105, 18.1%). Less than 5 patients received other AAs.

**Table 1:** Utilization of psychotropic drugs by class among 935 patients with Parkinson’s disease initiating pramipexole/ropinirole therapy. Please note that the total number of patients for each drug category is sometimes lower than the sum of all individuals listed for each treatment, since some patients were prescribed more than a single drug in each category.

*DRUG CATEGORIES*		Patients	%
**Glutamatergic modulators (GMs)**	Any GMs	264	100.0%
	Amantadine	227	86.0%
	Memantine	41	15.5%
	Topiramate	9	3.4%
	N-acetylcysteine	9	3.4%
	Acamprosate	0	0.0%
**Selective serotonin reuptake inhibitors (SSRIs)**	Any SSRIs	205	100.0%
	Citalopram / Escitalopram	149	72.7%
	Sertraline	61	29.8%
	Fluoxetine	29	14.1%
	Paroxetine	10	4.9%
	Fluvoxamine	2	1.0%
**Atypical antipsychotics (AAs)**	Any Aas	105	100.0%
	Quetiapine	96	91.4%
	Olanzapine	19	18.1%
	Aripiprazole	4	3.8%
	Risperidone	3	2.9%
	Ziprasidone	3	2.9%
	Clozapine	1	1.0%
	Paliperidone	1	1.0%
	Lurasidone	0	0.0%
	Asenapine	0	0.0%
	Iloperidone	0	0.0%
**Mood stabilizers (MSs)**	Any MSs	7	100.0%
	Lithium	3	42.9%
	Carbamazepine	3	42.9%
	Valproic acid/divalproex	1	14.2%
**Opioid receptor antagonists (ORAs)**	Any ORAs	0	0.0%
	Naltrexone	0	0.0%
	Nalmefene	0	0.0%

#### GMs

A total of 711 non-exposed patients were matched to the 264 exposed patients on age, gender, and duration of PPX/ROP. The ICD occurred at a significantly higher rate in the exposed group (20/264, 7.6%) compared to the non-exposed (10/711, 1.4%). The crude and adjusted OR were 4.99 (95% CI: 2.30–10.80) and 14.00 (95% CI: 3.58–54.4), respectively (Table 2).

**Table 2:** Characteristics and impulse control disorder risk of glutamatergic modulators (GMs) users compared to non users.

	Overall (N = 975, 100%)	Non-users (N = 711, 72.9%)	Users (N = 264, 27.1%)	P-value^a^
**Characteristics**				
Age, Mean (SD)	66.8 (9.2)	67.2 (8.9)	65.8 (10.0)	0.014
Sex–Males (%)	610 (62.6%)	448 (63.0%)	162 (61.4%)	0.655
Race—Caucasians (%)	875 (89.7%)	629 (88.5%)	246 (93.2%)	0.032
Marital Status–Married (%)	747 (76.6%)	536 (75.4%)	211(79.9%)	0.148
Levodopa / Cabergoline	241 (24.7%)	146 (20.5%)	95 (36.0%)	<0.001
**Comorbid Mental Illnesses**				
Dementia / Cognitive Disorders	149 (15.3%)	122 (17.2%)	27 (10.2%)	0.007
Depressive Disorders	216 (22.2%)	180 (25.3%)	36 (13.6%)	<0.001
Bipolar Disorders	31 (3.2%)	26 (3.7%)	5 (1.9%)	0.218
Schizophrenia / Other Psychotic Disorders	42 (4.3%)	36 (5.1%)	6 (2.3%)	0.074
Substance or Alcohol-Related Disorders	24 (2.5%)	21 (3.0%)	3 (1.1%)	0.160
**Impulse Control Disorder Risk**				
Number of events (%)	30 (3.1%)	10 (1.4%)	20 (7.6%)	<0.001
**Odds Ratios (95% Confidence Interval)**				
Crude			4.99 (2.30–10.80)	
Adjusted			14.00 (3.58–54.44)	

P-value^a^: X^2^-test was performed to compare crude incidences between groups in terms of Impulse control disorder and baseline characteristics.

#### SSRIs

After matching on age, gender, and duration of PPX/ROP at cohort entry, there were 554 patients in non-exposed group. Overall 23 patients (3.0%) developed ICD after initiation PPX/ROP. 13 cases (6.3%) were attributable to the concomitant use of SSRIs with PPX/ROP. Crude OR was 3.31 (95% CI: 1.44–7.65). The adjusted OR was 3.67 (95% CI: 1.07–12.60) after adjusting for comorbid mental disorder conditions, the use of levodopa/cabergoline, marital status, and race (Table 3).

**Table 3:** Characteristics and impulse control disorder risk of selective serotonin reuptake inhibitors (SSRIs) users compared to non users.

	Overall (N = 759, 100%)	Non-users (N = 554, 73.0%)	Users (N = 205, 27.0%)	P-value^a^
**Characteristics**				
Age, Mean (SD)	23 (3.0%)	10 (1.8%)	13 (6.3%)	0.014
Sex–Males (%)	67.6 (9.2)	68.1 (8.7)	66.3 (10.3)	0.655
Race—Caucasians (%)	455 (60.0%)	335 (60.5%)	120 (58.5%)	0.032
Marital Status–Married (%)	654 (86.2%)	471 (85.0%)	183 (89.3%)	0.148
Levodopa / Cabergoline	591 (77.9%)	434 (78.3%)	157 (76.6%)	<0.001
**Comorbid Mental Illnesses**				
Dementia / Cognitive Disorders	86 (11.3%)	67 (12.1%)	19 (9.3%)	0.007
Depressive Disorders	161 (21.2%)	99 (17.9%)	62 (30.2%)	<0.001
Bipolar Disorders	26 (3.4%)	24 (4.3%)	2 (1.0%)	0.218
Schizophrenia / Other Psychotic Disorders	47 (6.2%)	42 (7.6%)	5 (2.4%)	0.074
Substance or Alcohol-Related Disorders	21 (2.8%)	18 (3.3%)	3 (1.5%)	0.160
**Impulse Control Disorder Risk**				
Number of events (%)	23 (3.0%)	10 (1.8%)	13 (6.3%)	0.003
**Odds Ratios (95% Confidence Interval)**				
Crude			3.31 (1.44–7.65)	
Adjusted			3.67 (1.07–12.59)	

P-value^a^: X^2^-test was performed to compare crude incidences between groups in terms of Impulse control disorder and baseline characteristics.

#### AAs

After matching 105 exposed patients on age, gender, and duration of PPX/ROP at cohort entry, there were 295 patients in non-exposed group. In this AA model, ICD occurred at the lowest rate across all the three models, with 2.3% (9/400) of ICD incidence. The crude OR was 2.30 with 95% CI between 0.56 and 9.56. Adjusted OR could not be estimated due to problems with model convergence yielding unreliable OR estimate and confidence interval (Table 4).

**Table 4:** Characteristics and impulse control disorder risk of atypical antipsychotics (AAs) users compared to non users.

	Overall (N = 400, 100%)	Non-users (N = 295, 73.8%)	Users (N = 105, 26.3%)	P-value^a^
**Characteristics**				
Age, Mean (SD)	9 (2.3%)	5 (1.7%)	4 (3.8%)	0.250
Sex–Males (%)	70.2 (9.3)	70.4 (8.9)	69.6 (10.4)	0.038
Race—Caucasians (%)	261 (65.3%)	194 (65.8%)	67 (63.8%)	0.722
Marital Status–Married (%)	332 (83.0%)	235 (79.7%)	97 (92.4%)	0.002
Levodopa / Cabergoline	302 (75.5%)	225 (76.3%)	77 (73.3%)	0.597
**Comorbid Mental Illnesses**				
Dementia / Cognitive Disorders	51 (12.8%)	30 (10.2%)	21 (10.0%)	0.016
Depressive Disorders	49 (12.3%)	28 (9.4%)	21 (20.0%)	0.049
Bipolar Disorders	8 (2.0%)	2 (0.7%)	6 (5.7%)	0.005
Schizophrenia / Other Psychotic Disorders	16 (4.0%)	5 (1.7%)	11 (10.5%)	0.0003
Substance or Alcohol-Related Disorders	7 (1.8%)	4 (1.4%)	3 (2.9%)	0.385
**Impulse Control Disorder Risk**				
Number of events (%)	23 (3.0%)	10 (1.8%)	13 (6.3%)	0.003
**Odds Ratios (95% Confidence Interval)**				
Crude			2.30 (0.56–9.56)	
Adjusted			Not performed	

P-value^a^: X^2^-test was performed to compare crude incidences between groups in terms of Impulse control disorder and baseline characteristics.

## Discussion

The main result of the present study was that GMs, SSRIs, and AAs were not found to statistically decrease the risk of ICDs associated with concomitant PPX/ROP use in PD patients. Conversely, we observed a positive association between GM use and ICD incidence in a cohort matched by gender, age at PPX/ROP initiation, and duration of PPX/ROP therapy (7.6% vs 1.4%, P<0.0001). Strikingly, this association remained after controlling for baseline characteristics, including levodopa/cabergoline use, psychiatric comorbidities, and patient demographics.

Given that amantadine accounted for 90% of GM prescriptions in this population, these findings appear to be in keeping with previous reports documenting that this drug may increase ICD risk in PD patients. [31,32] However, as the effects of amantadine are not exclusively underpinned by glutamate modulation but also reflect its dopamine-enhancing properties, [26] caution should be advocated against any generalized conclusion on this collective drug class.

Both crude and adjusted ORs suggested that SSRI users were at higher risk for ICD development. The lack of effectiveness of SSRIs in reducing the risk of iatrogenic ICDs in PD is surprising, in consideration of previous clinical reports documenting the efficacy of citalopram and escitalopram in improving impulsive behaviors in PD and psychiatric patients. [33–35] It is worth noting that the results of this analysis should not be considered conclusive, due to the observational nature of our study and the existence of residual confounding factors that could not be included in our analysis. However, considering the ICD incidence rate of 6.3% in SSRI user group was more than three times greater than non-SSRI users, the resulting increase in the absolute risk due to residual confounding factors would need to be substantial to reverse the association toward protective. Overall, the observed incidence rates provide some assurance regarding a limited potential for the role of SSRIs in reducing clinically significant ICD risk even if general concerns about bias are considered. If confirmed, the lack of efficacy of SSRIs in reducing PPX/ROP-associated ICDs may signify that the neurotransmitter changes induced by these antidepressants are unlikely to affect the activation of dopamine receptors induced by DRTs. Albeit preliminary, these findings collectively suggest that several key medications that have been postulated to exert therapeutic properties for idiopathic ICDs may not have similar indications for their DRT-associated counterparts in PD. This discrepancy may reflect distinct pathophysiological underpinnings of these conditions. ICDs reflect the activation of D_3_ receptors by drugs, rather than by dopamine, and as such may be less amenable to treatments that can modify dopaminergic release in the ventral striatum. In alignment with this idea, we recently showed that, in animal models, the effects of PPX on risky decision making were not based on modifications of dopamine release but were likely related to changes in downstream signaling of postsynaptic dopamine receptors. [36] It is also possible that ICDs in PD patients may feature specific characteristics secondary to the neurodegenerative condition, such as modifications of dopamine signaling in the ventral striatum secondary to hypofunction of the mesolimbic system in vulnerable PD patients. In support of this idea, recent studies have documented that dopaminergic deficits in the projections from the ventral tegmental area to the nucleus accumbens may be a critical predisposing factor to PPX/ROP-associated ICDs in PD. [37]

Several limitations of this study should be acknowledged. First, the prevalence of ICDs in our PD population was estimated at 3%, a rate markedly smaller than that reported in prior studies (6–60%); [2] this discrepancy may suggest that using diagnostic codes may have a low sensitivity in capturing patients with ICD. By the same token, however, this approach has high specificity, and therefore minimizes the likelihood of inclusion of patients without an accurate ICD diagnosis. Under these conditions, relative risk estimates are generally considered unbiased. [38] Nevertheless, given that we cannot estimate the rate of undiagnosed ICDs in this population, the extent of potential outcome misclassification remains unknown. An alternative explanation to account for this rate discrepancy may lie in the specific sociodemographic and cultural characteristics of Utah, which is the US state with the heaviest legal restrictions on gambling and betting. Thus, local PD patients may have fewer avenues to engage in these activities, ultimately resulting in lower ICD incidence.

Second, although our matching and adjustment analyses balanced patients on key risk factors -such as patient demographics, duration of PPX/ROP, and the use of levodopa/cabergoline -, the possibility of confounding bias cannot be entirely ruled out. For example, only 30% of patients treated with a SSRI had a comorbid diagnosis of depression at the time of SSRI initiation. Given that SSRIs are primarily prescribed for the treatment of depression and other psychiatric disorders, we anticipate a higher rate of undiagnosed depressive disorders in new SSRI users. An analogous argument could be used for amantadine, since this drug is used as a treatment for dyskinesias and motor fluctuations in PD, and these complications are robustly associated with ICDs. [39,40] However, our study controlled for PPX/ROP use in the study design and use of levodopa in regression model, which subsequently balanced patients with regard to dyskinesias.

Third, due to limitations in statistical power, we could not analyze other treatments for ICDs, such as MSs, ORAs, and voltage-dependent calcium channel inhibitors (e.g., pregabalin and gabapentin). [41] Furthermore, even the three main classes of drugs analyzed are rather heterogeneous in terms of mechanisms, leaving the possibility open that specific drugs in each category may have specific efficacy in reducing ICD severity. In the case of AAs, for example, our results were skewed by effects of quetiapine, which accounted for more than 90% of this group. While the use of this drug in PD is warranted by several reports showing its safety and antipsychotic efficacy in PD, [42–44] this drug is poorly effective in blocking D_3_ dopamine receptors, [45,46] which are considered to play a key role in DRT-associated ICDs. [37] Conversely, AAs with relatively high affinity for D_3_ receptors, such as risperidone [47], were under-represented in our sample, in alignment with warnings on the extrapyramidal adverse events associated with this drug. [48]

Finally, the small number of AA-treated patients developing ICDs in our population did not allow us to adjust for potential confounding factors. Future studies with adequate sample sizes will be needed to address these critical issues.

These caveats notwithstanding, the present findings suggest that GMs and SSRIs may not yield clinically meaningful therapeutic effects for DRT-associated ICDs in PD patients. This result awaits confirmation in causal analyses of observational data with adequate sample size, which will allow for controlling potential confounding factors while improving statistical power. For example, future analyses may be restricted to patients with indications for a specific drug and with comprehensive coverage of health care services, permitting a broader selection of variables for adjustment. It is also worth noting that the approach used in this study holds promise as a powerful data-mining methodology for drug discovery, which could be successfully coupled to screening in animal models of DRT-associated impulsivity. In this perspective, our group recently developed a rat model of probability discounting specifically optimized to capture the increase in probability discounting following PPX treatment. [36]

## Supporting information

S1 Table ICD 9/10 –CM codes by mental disorder type. (DOCX)

## References

[1] Weintraub D, Koester J, Potenza MN, Siderowf AD, Stacy M, Voon V, et al Impulse control disorders in Parkinson disease: A cross-sectional study of 3090 patients. Arch Neurol [Internet]. 2010 May 1 [cited 2019 Apr 4];67(5):589–95. Available from: http://archneur.jamanetwork.com/article.aspx?doi=10.1001/archneurol.2010.65 20457959 10.1001/archneurol.2010.65

[2] Molde H, Moussavi Y, Kopperud ST, Erga AH, Hansen AL, Pallesen S. Impulse-control disorders in Parkinson’s Disease: A meta-analysis and review of case-control studies. Front Neurol. 2018 5;9(MAY):330. 29872418 10.3389/fneur.2018.00330 PMC5972311

[3] Holloway RG. Pramipexole vs levodopa as initial treatment for Parkinson Disease: A 4-year randomized controlled trial. Arch Neurol [Internet]. 2004 7 1 [cited 2019 Apr 23];61(7):1044–53. Available from: http://www.ncbi.nlm.nih.gov/pubmed/15262734 10.1001/archneur.61.7.1044 15262734

[4] Korczyn AD, Brooks DJ, Brunt ER, Poewe WH, Rascol O, Stocchi F. Ropinirole versus bromocriptine in the treatment of early Parkinson’s disease: A 6-month interim report of a 3-year study. Mov Disord [Internet]. 1998 1 1 [cited 2019 Apr 23];13(1):46–51. Available from: http://doi.wiley.com/10.1002/mds.870130112 9452325 10.1002/mds.870130112

[5] Weddle KE, Guo JJ, Wigle PR, Ajayi FO. Assocation between impulse control disorders and the use of dopamine agonists. Pharmacoepidemiol Drug Saf [Internet]. 2011;20(PG-S196-S197):S196–7. Available from: http://www.embase.com/search/results?subaction=viewrecord&from=export&id=L70665300 NS -

[6] Aguirre C, García M, Lertxundi U. Impulsive control disorders associated with dopamine agonists drugs: Analysis of cases reported in the European pharmacovigilance database (eudravigilance). Basic Clin Pharmacol Toxicol [Internet]. 2016;119(PG-40):40 Available from: http://www.embase.com/search/results?subaction=viewrecord&from=export&id=L617433913 NS -

[7] Moore TJ, Glenmullen J, Mattison DR. Reports of pathological gambling, hypersexuality, and compulsive shopping associated with dopamine receptor agonist drugs. JAMA Intern Med [Internet]. 2014 12 1 [cited 2019 Apr 4];174(12):1930–3. Available from: http://www.ncbi.nlm.nih.gov/pubmed/25329919 10.1001/jamainternmed.2014.5262 25329919

[8] Marques A, Durif F, Fernagut PO. Impulse control disorders in Parkinson’s disease. J Neural Transm. 2018;125(8):1299–312. 10.1007/s00702-018-1870-8 29511827

[9] Grall-Bronnec M, Victorri-Vigneau C, Donnio Y, Leboucher J, Rousselet M, Thiabaud E, et al Dopamine Agonists and Impulse Control Disorders: A Complex Association. Drug Saf [Internet]. 2018 1 [cited 2018 Oct 1];41(1):19–75. Available from: http://www.ncbi.nlm.nih.gov/pubmed/28861870 10.1007/s40264-017-0590-6 28861870 PMC5762774

[10] Nirenberg MJ. Dopamine Agonist Withdrawal Syndrome: Implications for Patient Care. Drugs Aging. 2013 8;30(8):587–92. 10.1007/s40266-013-0090-z 23686524

[11] Vilas D, Pont-Sunyer C, Tolosa E. Impulse control disorders in Parkinson’s disease. Parkinsonism Relat Disord [Internet]. 2012 1 1 [cited 2019 Apr 26];18:S80–4. Available from: https://www.sciencedirect.com/science/article/pii/S1353802011700268?via%3Dihub 10.1016/S1353-8020(11)70026-8 22166463

[12] Kim SW, Grant JE, Adson DE, Shin YC, Zaninelli R. A double-blind placebo-controlled study of the efficacy and safety of paroxetine in the treatment of pathological gambling. J Clin Psychiatry [Internet]. 2002 6 [cited 2019 Apr 16];63(6):501–7. Available from: http://www.ncbi.nlm.nih.gov/pubmed/12088161 10.4088/jcp.v63n0606 12088161

[13] Grant JE, Potenza MN. Escitalopram treatment of pathological gambling with co-occurring anxiety: an open-label pilot study with double-blind discontinuation. Int Clin Psychopharmacol [Internet]. 2006 7 [cited 2019 Apr 16];21(4):203–9. Available from: http://www.ncbi.nlm.nih.gov/pubmed/16687991 10.1097/00004850-200607000-00002 16687991

[14] Coccaro EF, Lee RJ, Kavoussi RJ. A Double-Blind, Randomized, Placebo-Controlled Trial of Fluoxetine in Patients With Intermittent Explosive Disorder. J Clin Psychiatry [Internet]. 2009 5 15 [cited 2019 Apr 16];70(5):653–62. Available from: http://www.psychiatrist.com/abstracts/abstracts.asp?abstract=200905/050903.htm 10.4088/JCP.08m04150 19389333

[15] Van den Eynde F, Senturk V, Naudts K, Vogels C, Bernagie K, Thas O, et al Efficacy of Quetiapine for Impulsivity and Affective Symptoms in Borderline Personality Disorder. J Clin Psychopharmacol [Internet]. 2008 4 [cited 2019 Apr 26];28(2):147–55. Available from: http://www.ncbi.nlm.nih.gov/pubmed/18344724 10.1097/JCP.0b013e318166c4bf 18344724

[16] Fong T, Kalechstein A, Bernhard B, Rosenthal R, Rugle L. A double-blind, placebo-controlled trial of olanzapine for the treatment of video poker pathological gamblers. Pharmacol Biochem Behav [Internet]. 2008 5 [cited 2019 Apr 29];89(3):298–303. Available from: http://www.ncbi.nlm.nih.gov/pubmed/18261787 10.1016/j.pbb.2007.12.025 18261787

[17] Coskun F, Akca OF. Treatment of Intermittent Explosive Disorder With Carbamazepine. Clin Neuropharmacol. 2018 3;41(2):82–3. 10.1097/WNF.0000000000000276 29533363

[18] Pallanti S, Quercioli L, Sood E, Hollander E. Lithium and valproate treatment of pathological gambling: a randomized single-blind study. J Clin Psychiatry [Internet]. 2002 7 [cited 2019 Apr 25];63(7):559–64. Available from: http://www.ncbi.nlm.nih.gov/pubmed/12143910 10.4088/jcp.v63n0704 12143910

[19] Papay K, Xie SX, Stern M, Hurtig H, Siderowf A, Duda JE, et al Naltrexone for impulse control disorders in Parkinson disease. Neurology. 2014 8;83(9):826–33. 10.1212/WNL.0000000000000729 25037206 PMC4155050

[20] Grant JE, Potenza MN, Hollander E, Cunningham-Williams R, Nurminen T, Smits G, et al Multicenter Investigation of the Opioid Antagonist Nalmefene in the Treatment of Pathological Gambling. Am J Psychiatry [Internet]. 2006 2 [cited 2019 Apr 25];163(2):303–12. Available from: http://www.ncbi.nlm.nih.gov/pubmed/16449486 10.1176/appi.ajp.163.2.303 16449486

[21] Pettorruso M, De Risio L, Martinotti G, Di Nicola M, Ruggeri F, Conte G, et al Targeting the Glutamatergic System to Treat Pathological Gambling: Current Evidence and Future Perspectives. Biomed Res Int. 2014; 10.1155/2014/109786 PMC4075088 25013755

[22] Grant JE, Kim SW, Odlaug BL. N-Acetyl Cysteine, a Glutamate-Modulating Agent, in the Treatment of Pathological Gambling: A Pilot Study. Biol Psychiatry [Internet]. 2007 9 15 [cited 2019 Apr 27];62(6):652–7. Available from: http://www.ncbi.nlm.nih.gov/pubmed/17445781 10.1016/j.biopsych.2006.11.021 17445781

[23] Driver-Dunckley E, Samanta J, Stacy M. Pathological gambling associated with dopamine agonist therapy in Parkinson’s disease. Neurology [Internet]. 2003 8 12 [cited 2019 Apr 26];61(3):422–3. Available from: http://www.ncbi.nlm.nih.gov/pubmed/12913220 10.1212/01.wnl.0000076478.45005.ec 12913220

[24] Thomas A, Bonanni L, Gambi F, Di Iorio A, Onofrj M. Pathological gambling in Parkinson disease is reduced by amantadine. Ann Neurol [Internet]. 2010 9 [cited 2019 Apr 26];68(3):400–4. Available from: http://www.ncbi.nlm.nih.gov/pubmed/20687121 10.1002/ana.22029 20687121

[25] Vittinghoff E, McCulloch CE. Relaxing the Rule of Ten Events per Variable in Logistic and Cox Regression. Am J Epidemiol [Internet]. 2007 1 12 [cited 2019 May 11];165(6):710–8. Available from: https://academic.oup.com/aje/article-lookup/doi/10.1093/aje/kwk052 17182981 10.1093/aje/kwk052

[26] Weiss HD, Marsh L. Impulse control disorders and compulsive behaviors associated with dopaminergic therapies in Parkinson disease. Neurol Clin Pract [Internet]. 2012 12 [cited 2019 Apr 24];2(4):267 Available from: http://www.ncbi.nlm.nih.gov/pubmed/23634371 10.1212/CPJ.0b013e318278be9b 23634371 PMC3613210

[27] McElroy SL, Pope HG, Keck PE, Hudson JI, Phillips KA, Strakowski SM. Are impulse-control disorders related to bipolar disorder? Compr Psychiatry [Internet]. 1996 7 1 [cited 2019 Apr 24];37(4):229–40. Available from: https://www.sciencedirect.com/science/article/pii/S0010440X96900012?via%3Dihub 10.1016/s0010-440x(96)90001-2 8826686

[28] Hoptman MJ. Impulsivity and aggression in schizophrenia: a neural circuitry perspective with implications for treatment. CNS Spectr [Internet]. 2015 6 [cited 2019 Apr 24];20(3):280–6. Available from: http://www.ncbi.nlm.nih.gov/pubmed/25900066 10.1017/S1092852915000206 25900066 PMC4441843

[29] Bidzan L, Bidzan M, Pąchalska M. Aggressive and impulsive behavior in Alzheimer’s disease and progression of dementia. Med Sci Monit. 2012;18(3):CR182–9. 10.12659/MSM.882523 22367129 PMC3560760

[30] Lejoyeux M, Arbaretaz M, McLoughlin M, Adiès J. Impulse control disorders and depression. J Nerv Ment Dis. 2002;190(5):310–4. 10.1097/00005053-200205000-00007 12011611

[31] Weintraub D, Sohr M, Potenza MN, Siderowf AD, Stacy M, Voon V, et al Amantadine use associated with impulse control disorders in Parkinson disease in cross-sectional study. Ann Neurol [Internet]. 2010 12 [cited 2019 Apr 27];68(6):963–8. Available from: http://doi.wiley.com/10.1002/ana.22164 21154480 10.1002/ana.22164

[32] Walsh RA, Lang AE. Multiple impulse control disorders developing in Parkinson’s disease after initiation of amantadine [Internet]. Vol. 27, Movement Disorders. John Wiley & Sons, Ltd; 2012 [cited 2019 May 15]. p. 326–7. Available from: http://doi.wiley.com/10.1002/mds.23964 21954056 10.1002/mds.23964

[33] Weintraub D, Taraborelli D, Morales KH, Duda JE, Katz IR, Stern MB. Escitalopram for Major Depression in Parkinson’s Disease: An Open-Label, Flexible-Dosage Study. J Neuropsychiatry Clin Neurosci. 2014;18(3):377–83. 10.1176/appi.neuropsych.18.3.377 PMC1761053 16963587

[34] Dell’Osso B, Hadley S, Allen A, Baker B, Chaplin WF, Hollander E. Escitalopram in the treatment of impulsive-compulsive internet usage disorder: An open-label trial followed by a double-blind discontinuation phase. J Clin Psychiatry. 2008;69(3):452–6. 10.4088/jcp.v69n0316 18312057

[35] Zimmerman M, Breen RB, Posternak MA. An open-label study of citalopram in the treatment of pathological gambling. J Clin Psychiatry. 2002;63(1):44–8. 10.4088/jcp.v63n0109 11838625

[36] Pes R, Godar SC, Fox AT, Burgeno LM, Strathman HJ, Jarmolowicz DP, et al Pramipexole enhances disadvantageous decision-making: Lack of relation to changes in phasic dopamine release. Neuropharmacology [Internet]. 2017 3 1 [cited 2019 Apr 27];114:77–87. Available from: http://www.ncbi.nlm.nih.gov/pubmed/27889491 10.1016/j.neuropharm.2016.11.014 27889491 PMC5241163

[37] Seeman P. Parkinson’s disease treatment may cause impulse-control disorder via dopamine D3 receptors. Synapse. 2015;69(4):183–9. 10.1002/syn.21805 25645960

[38] Schneeweiss S, Avorn J. A review of uses of health care utilization databases for epidemiologic research on therapeutics. J Clin Epidemiol [Internet]. 2005 4 1 [cited 2019 May 14];58(4):323–37. Available from: https://www.sciencedirect.com/science/article/pii/S0895435604002987 10.1016/j.jclinepi.2004.10.012 15862718

[39] Thomas A, Iacono D, Luciano AL, Armellino K, Di Iorio A, Onofrj M. Duration of amantadine benefit on dyskinesia of severe Parkinson’s disease. J Neurol Neurosurg Psychiatry [Internet]. 2004 1 [cited 2019 May 14];75(1):141–3. Available from: http://www.ncbi.nlm.nih.gov/pubmed/14707325 14707325 PMC1757492

[40] Biundo R, Weis L, Abbruzzese G, Calandra-Buonaura G, Cortelli P, Jori MC, et al Impulse control disorders in advanced Parkinson’s disease with dyskinesia: The ALTHEA study. Mov Disord. 2017;32(11):1557–65. 10.1002/mds.27181 28960475

[41] Schreiber L, Odlaug BL, Grant JE. Impulse control disorders: updated review of clinical characteristics and pharmacological management. Front psychiatry [Internet]. 2011 [cited 2018 Oct 1];2:1 Available from: http://www.ncbi.nlm.nih.gov/pubmed/21556272 10.3389/fpsyt.2011.00001 21556272 PMC3089999

[42] Molho ES, Factor SA. Parkinson’s disease: The treatment of drug-induced hallucinations and psychosis. Curr Neurol Neurosci Rep. 2001;1(4):320–328. 10.1007/s11910-001-0085-8 11898537

[43] Friedman JH. Atypical antipsychotics in the EPS-vulnerable patient. Psychoneuroendocrinology. 2003;28:39–51. 10.1016/s0306-4530(02)00111-7 12504071

[44] Seppi K, Weintraub D, Coelho M, et al The Movement Disorder Society Evidence-Based Medicine Review Update: Treatments for the non-motor symptoms of Parkinson’s disease. Mov Disord. 2011;26(S3):S42–S80. 22021174 10.1002/mds.23884 PMC4020145

[45] Burstein ES, Ma J, Wong S, et al Intrinsic Efficacy of Antipsychotics at Human D2, D3, and D4 Dopamine Receptors: Identification of the clozapine metabolite N-desmethylclozapine as a D2/D3 partial agonist. J Pharmacol Exp Ther. 2005;315(3):1278–1287 10.1124/jpet.105.092155 16135699

[46] Mizrahi R, Agid O, Borlido C, et al Effects of antipsychotics on D3 receptors: A clinical PET study in first episode antipsychotic naive patients with schizophrenia using [11C]-(+)-PHNO. Schizophr Res. 2011;131(1–3):63–68. 10.1016/j.schres.2011.05.005 21684721

[47] Leysen JE, Janssen PM, Megens AA, Schotte A. Risperidone: a novel antipsychotic with balanced serotonin-dopamine antagonism, receptor occupancy profile, and pharmacologic activity. J Clin Psychiatry. 1994 5;55 Suppl:5–12. 7520908

[48] Fernandez HH, Trieschmann ME, Friedman JH. Treatment of psychosis in Parkinson’s disease. Drug Saf. 2003;26(9):643–659. 10.2165/00002018-200326090-00004 12814332

