# Supplementary material for: What drugs modify the risk of iatrogenic impulse-control disorders in Parkinson’s disease? A preliminary pharmacoepidemiologic study
Source: PLoS One. 2020 Jan 7;15(1):e0227128. doi: 10.1371/journal.pone.0227128 (PMC6946157; doi:10.1371/journal.pone.0227128)
Supplement: S1 Table — (DOCX) [file pone.0227128.s001.docx]

**S1 Table 1. ICD 9/10 –CM codes by mental disorder type**

| **Mental disorder** | **ICD 9 CM code** | **ICD 10 CM code** |
| --- | --- | --- |
| Delirium/Dementia/Amnestic/Other Cognitive Disorders | 290.0, 290.10, 290.11, 290.12, 290.13, 290.20, 290.21, 290.3, 290.40, 290.41, 290.42, 290.43, 290.8, 290.9, 293.0, 293.1, 294.0, 294.1, 294.10, 294.11, 294.20, 294.21, 294.8, 294.9, 310.0, 310.2, 310.8, 310.81, 31089, 310.9, 331.0, 331.1, 331.11, 331.19, 331.2, 331.82, 797 | F0150, F0151, F0280, F0281, F0390, F0391, F04, F05, F070, F0781, F0789, F079, F09, F482, G300, G301, G308, G309, G3101, G3109, G311, G3183, R4181, R54 |
| Depressive Disorders | 293.83, 296.20, 296.21, 296.22, 296.23, 296.24, 296.25, 296.26, 296.30, 296.31, 296.32, 296.33, 296.34, 296.35, 296.36, 300.4, 311 | F0630, F0631, F0632, F320, F321, F322, F323, F324, F325, F328, F3281, F3289,  F329, F330, F331, F332, F333, F3340, F3341, F3342, F338, F339, F341 |
| Bipolar Disorders | 296.00, 296.01, 296.02, 296.03, 296.04, 296.05, 296.06, 296.10, 296.11, 296.12, 296.13, 296.14, 296.15, 296.16, 296.40, 296.41, 296.42, 296.43, 296.44, 296.45, 296.46, 296.50, 296.51, 296.52, 296.53, 296.54, 296.55, 296.56, 296.60, 296.61, 296.62, 296.63, 296.64, 296.65, 296.66, 296.7, 296.80, 296.81, 296.82, 296.89, 296.90, 296.99 | F0633, F0634, F3010, F3011, F3012, F3013, F302, F303, F304, F308, F309, F310, F3110, F3111, F3112, F3113, F312, F3130, F3131, F3132, F314, F315, F3160, F3161, F3162, F3163, F3164, F3170, F3171, F3172, F3173, F3174, F3175, F3176, F3177, F3178, F3181, F3189, F319, F340, F348, F3481, F3489, F349, F39 |
| Schizophrenia and Other Psychotic Disorders | 293.81, 293.82, 295.00, 295.01, 295.02, 295.03, 295.04, 295.05, 295.10, 295.11, 295.12, 295.13, 295.14, 295.15, 295.20, 295.21, 295.22, 295.23, 295.24, 295.25, 295.30, 295.31, 295.32, 295.33, 295.34, 295.35, 295.40, 295.41, 295.42, 295.43, 295.44, 295.45, 295.50, 295.51, 295.52, 295.53, 295.54, 295.55, 295.60, 295.61, 295.62, 295.63, 295.64, 295.65, 295.70, 295.71, 295.72, 295.73, 295.74, 295.75, 295.80, 295.81, 295.82, 295.83, 295.84, 295.85, 295.90, 295.91, 295.92, 295.93,  295.94, 295.95, 297.0, 297.1, 297.2, 297.3, 297.8, 297.9, 298.0, 298.1, 298.2, 298.3, 298.4, 298.8, 298.9 | F060, F062, F200, F201, F202, F203, F205, F2081, F2089, F209, F21, F22, F23, F24, F250, F251, F258, F259, F28, F29 |
| Substance-Related Disorders | 291.0, 291.1, 291.2, 291.3, 2914, 291.5, 291.8, 291.81, 291.82, 291.89, 291.9, 303.00, 303.01, 303.02, 30303, 303.90, 303.91, 303.92, 303.93, 305.00, 305.01, 305.02, 305.03, 357.5, 425.5, 535.3, 535.30, 53531, 5710, 5711, 571.2, 571.3, 760.71, 980.0; 292.0, 292.11, 292.12, 292.2, 292.81, 292.82, 292.83, 292.84, 292.85, 292.89, 292.9, 304.00, 304.01, 304.02, 304.03, 304.10, 304.11, 304.12, 304.13, 304.20, 304.21, 304.22, 304.23, 304.30, 304.31, 304.32, 304.33, 304.40, 304.41, 304.42, 304.43, 304.50, 304.51, 304.52, 304.53, 304.60, 304.61, 304.62, 304.63, 304.70, 304.71, 304.72, 304.73, 304.80, 304.81, 304.82, 304.83, 304.90, 304.91, 304.92, 304.93, 305.20, 305.21, 305.22, 305.23, 305.30, 305.31, 305.32, 305.33, 305.40, 305.41, 305.42, 305.43, 305.50, 305.51, 305.52, 305.53, 305.60, 305.61, 305.62, 305.63, 305.70, 305.71, 305.72, 305.73, 305.80, 305.81, 305.82, 305.83, 305.90, 305.91, 305.92, 305.93, 648.30, 648.31, 648.32, 648.33, 648.34, 655.50, 655.51, 655.53, 760.72, 760.73, 760.75, 779.5, 965.00, 965.01, 965.02, 965.09, V65.42 | F10.10, F10.11, F10.120, F10.121, F10.129, F10.14, F10.150, F10.151, F10.159, F10.180, F10.181, F10.182, F10.188, F10.19, F10.20, F10.21, F10.220, F10.221, F10.229, F10.230,  F10.231, F10.232, F10.239, F10.24, F10.250, F10.251, F10.259, F10.26, F10.27, F10.280, F10.281, F10.282, F10.288, F10.29, F10.920, F10.921, F10.929, F10.94, F10.950, F10.951,  F10.959, F10.96, F10.97, F10.980, F10.981, F10.982, F10.988, F10.99, G62.1, I42.6, K29.20, K29.21, K70.0, K70.10, K70.11, K70.2, K70.30, K70.31, K70.40, K70.9, O99.310, O99.311, O99.312, O99.313, O99.314, O99.315, P04.3, Q86.0; F1110, F1111, F11120, F11121, F11122, F11129, F1114, F11150, F11151, F11159, F11181, F11182, F11188, F1119, F1120, F1121, F11220, F11221, F11222, F11229, F1123, F1124, F11250, F11251, F11259, F11281, F11282, F11288, F1129, F1190, F11920, F11921, F11922, F11929, F1193, F1194, F11950, F11951, F11959, F11981, F11982, F11988, F1199, F1210, F1211, F12120, F12121, F12122, F12129, F12150, F12151, F12159, F12180, F12188, F1219, F1220, F1221, F12220,  F12221, F12222, F12229, F12250, F12251, F12259, F12280, F12288, F1229, F1290, F12920, F12921, F12922, F12929, F12950, F12951, F12959, F12980, F12988, F1299, F1310, F1311,  F13120, F13121, F13129, F1314, F13150, F13151, F13159, F13180, F13181, F13182, F13188, F1319, F1320, F1321, F13220, F13221, F13229, F13230, F13231, F13232, F13239, F1324, F13250, F13251, F13259, F1326, F1327, F13280, F13281, F13282, F13288, F1329, F1390, F13920, F13921, F13929, F13930, F13931, F13932, F13939, F1394, F13950, F13951, F13959, F1396, F1397, F13980, F13981,  F13982, F13988, F1399, F1410, F1411, F14120, F14121, F14122, F14129, F1414, F14150, F14151, F14159, F14180, F14181, F14182, F14188, F1419, F1420, F1421, F14220, F14221, F14222, F14229,  F1423, F1424, F14250, F14251, F14259, F14280, F14281, F14282, F14288, F1429, F1490, F14920, F14921, F14922, F14929, F1494, F14950, F14951, F14959, F14980, F14981, F14982, F14988, F1499, F1510, F1511, F15120, F15121, F15122, F15129,  F1514, F15150, F15151, F15159, F15180, F15181, F15182, F15188, F1519, F1520, F1521, F15220,  F15221, F15222, F15229, F1523, F1524, F15250, F15251, F15259, F15280, F15281, F15282, F15288, F1529, F1590, F15920, F15921, F15922, F15929, F1593, F1594, F15950, F15951, F15959, F15980, F15981, F15982, F15988, F1599, F1610, F1611, F16120, F16121, F16122, F16129, F1614, F16150, F16151, F16159, F16180, F16183, F16188, F1619, F1620, F1621, F16220, F16221, F16229, F1624, F16250, F16251, F16259, F16280, F16283, F16288, F1629, F1690, F16920, F16921,  F16929, F1694, F16950, F16951, F16959, F16980, F16983, F16988, F1699, F17200, F17201, F17203,  F17208, F17209, F17210, F17211, F17213, F17218, F17219, F17220, F17221, F17223, F17228, F17229, F17290, F17291, F17293, F17298, F17299, F1810, F1811, F18120, F18121, F18129, F1814, F18150, F18151, F18159, F1817, F18180, F18188, F1819, F1820, F1821, F18220, F18221, F18229, F1824, F18250, F18251, F18259, F1827, F18280, F18288, F1829, F1890, F18920, F18921, F18929, F1894, F18950, F18951, F18959, F1897, F18980, F18988, F1899, F1910, F1911, F19120, F19121, F19122, F19129, F1914, F19150, F19151, F19159, F1916, F1917, F19180, F19181, F19182, F19188, F1919, F1920, F1921, F19220, F19221, F19222, F19229, F19230, F19231, F19232, F19239, F1924, F19250, F19251,  F19259, F1926, F1927, F19280, F19281, F19282, F19288, F1929, F1990, F19920, F19921, F19922, F19929, F19930, F19931, F19932, F19939, F1994, F19950, F19951, F19959, F1996, F1997, F19980, F19981, F19982, F19988, F1999, F550, F551, F552, F553, F554, F558, O355XX0, O355XX1, O355XX2, O355XX3, O355XX4, O355XX5, O355XX9, O99320, O99321, O99322, O99323, O99324, O99325, P0441, P0449, P961, P962, T400X1A, T400X1D, T400X1S, T400X3A, T400X3D, T400X3S, T400X4A, T400X4D, T400X4S, T400X5A, T400X5D, T400X5S, T400X6A, T400X6D, T400X6S, T401X1A, T401X1D, T401X1S, T401X3A, T401X3D, T401X3S, T401X4A, T401X4D, T401X4S, T401X5A, T401X5D, T401X5S,  T405X1A, T405X1D, T405X1S, T405X3A, T405X3D, T405X3S, T405X4A, T405X4D,  T405X4S, T405X5A, T405X5D, T405X5S,  T405X6A, T405X6D, T405X6S, T407X1A, T407X1D, T407X1S, T407X3A, T407X3D,  T407X3S, T407X4A, T407X4D, T407X4S,  T407X5A, T407X5D, T407X5S, T407X6A,  T407X6D, T407X6S, T408X1A, T408X1D,  T408X1S, T408X3A, T408X3D, T408X3S,  T408X4A, T408X4D, T408X4S, T408X5A,  T408X5D, T408X5S, T40901A, T40901D,  T40901S, T40903A, T40903D, T40903S,  T40904A, T40904D, T40904S, T40905A,  T40905D, T40905S, T40906A, T40906D,  T40906S, T40991A, T40991D, T40991S,  T40993A, T40993D, T40993S, T40994A, T40994D, T40994S, T40995A, T40995D,  T40995S, T40996A, T40996D, T40996S |
