# Supplementary material for: What drugs modify the risk of iatrogenic impulse-control disorders in Parkinson’s disease? A preliminary pharmacoepidemiologic study
Source: PLoS One. 2020 Jan 7;15(1):e0227128. doi: 10.1371/journal.pone.0227128 (PMC6946157; doi:10.1371/journal.pone.0227128)
Supplement: Attachment — Submitted filename: Response to Reviewers.docx [file pone.0227128.s002.docx]

**Response to Reviewer 1.**

The present study investigates the potential of atypical antipsychotics (AAs), selective serotonin reuptake inhibitors (SSRIs), and glutamatergic modulators in Parkinson’s disease (PD) patients treated with pramipexole (PPX) and ropinirole (ROP) to prevent impulse control disorders (ICDs). Results have the potential to enhance our understanding of the respective side effects.

We would like to thank the Reviewer for his/her kind comments.

Drugs subsumed as antipsychotics are rather heterogeneous with respect to efficiency and potential side effects. Quetiapine, which was used in the majority of patients, has profound anticholinergic side effects but a rather moderate antipsychotic efficiency. Other compounds such as clozapine were only received by some patients. These points should at least be mentioned when the efficacy of "antipsychotics" is discussed.

We agree with the Reviewer. In this revised version, this limitation has been clearly discussed (lines 326-341). The use of quetiapine in our population is likely in line with evidence showing that, unlike other antipsychotics, quetiapine has a good safety profile for PD patients.

From a clinical standpoint, cognitive impairment and dementia are common comorbidities in PD and may increase the vulnerability of patients towards side effects. Memantine is often prescribed in this situation.

We agree with the Reviewer that PD patients with comorbid dementia or cognitive impairment are more likely to receive a memantine prescription and develop impulse control disorders. Thus, we included dementia/cognitive impairment as one of the covariates for the logistic regression models.

**Response to Reviewer 2.**

In the abstract, specify the number of subjects and the results of the logistic regression.

As advised, we have added the number of patients included in our analysis (line 39), as well as the odds ratios computed from the logistic regression in the results section of the abstract (lines 41-44).

The figure with OR crude and adjusted can be eliminated and the info included in the text

In agreement with this recommendation, we have removed the figure and corresponding text from the manuscript.

Why did you include the table with ICD codes? Are those diagnoses from the medical records of the patients included in the study? If yes, you should include also how many times that diagnosis has been present in your cohort.

The table includes the list of diagnosis codes that were considered to identify patients with a respective medical condition for covariate adjustment in our logistic regression. The use of the diagnoses listed in the table are explained on lines 166-170.

In table 4, did you use chi-square or logistic regression to test the effect of AP?

We used a logistic regression to test the effect of AP, as described on lines 159-161.

What covariate did you use to adjust for SSRI in table 3? Why did you not use the same covariates in the antipsychotic analysis?

As shown in table 3, age, sex, race, marital status, the use of levodopa/cabergoline, and several psychiatric conditions were included as covariates. We included the same covariates in the atypical antipsychotic (AA) analysis; however, there was an issue regarding model convergence yielding an unreliable estimate of the AA effect. For this reason, we did not report an adjusted odds ratio for the AA model. This was explained on lines 242-244.

How many glutamate modulators were administered in this cohort?

A total of 264 out of 935 patients received a glutamatergic medication such as amantadine, memantine, topiramate, N-acetylcysteine, and acamprosate. The break-down numbers of patients by type of glutamatergic medications are shown in Table 1.

In Table 1, the numbers do not match if you consider monotherapy; were there patients taking 2 SSRI?

Yes. There were patients who took two or more SSRIs or other drugs listed in the same category. This is now explained in detail in Table 1 legend.

The alpha of 0.05 does not seem very conservative; comment on it.

While we understand and appreciate this concern, our original decision to set alpha at P<0.05 was made in consideration of the preliminary, exploratory nature of these studies, which were specifically designed to identify potential negative associations between some drugs and PPX- and ROP-induced ICDs. At any rate, all p values were listed in the tables. Given that 1) use of corrections for multiple comparisons remains controversial with several authors, and 2) using a more conservative criterion for alpha would not affect our negative conclusions, we decided to maintain alpha at 0.05.

Were the patients in this cohort on anticholinergic meds?

Yes, but these treatments did not modify any of the associations reported in our study.

Can you calculate specificity and sensitivity of the logistic regression model?

The use of logistic regression has a different implication for evaluating associations than they have in prediction modeling. Our study focuses on evaluation of associations between the use of a drug and the risk of impulse control disorders not developing a prediction model. Generally, in the evaluation of associations between an exposure and an outcome, specificity and sensitivity are not discussed. They are used to present model performance in prediction modeling.
